# Two-Stage Interpretation of Changes in TEER of Intestinal Epithelial Layers Protected by Adhering Bifidobacteria During *E. coli* Challenges

**DOI:** 10.3389/fmicb.2020.599555

**Published:** 2020-11-19

**Authors:** Lu Yuan, Henny C. van der Mei, Henk J. Busscher, Brandon W. Peterson

**Affiliations:** Department of Biomedical Engineering, University Medical Center Groningen, University of Groningen, Groningen, Netherlands

**Keywords:** TEER, probiotics, intestinal microflora, tight-junctions, barrier integrity, real-time monitoring

## Abstract

Mechanisms of gastrointestinal protection by probiotic bacteria against infection involve amongst others, modulation of intestinal epithelial barrier function. Trans-epithelial electrical resistance (TEER) is widely used to evaluate cellular barrier functions. Here, we developed a two-stage interpretative model of the time-dependence of the TEER of epithelial layers grown in a transwell during *Escherichia coli* challenges in the absence or presence of adhering bifidobacteria. *E. coli* adhesion in absence or presence of adhering bifidobacteria was enumerated using selective plating. After 4–8 h, *E. coli* challenges increased TEER to a maximum due to bacterial adhesion and increased expression of a tight-junction protein [zonula occludens-1 (ZO-1)], concurrent with a less dense layer structure, that is indicative of mild epithelial layer damage. Before the occurrence of a TEER-maximum, decreases in electrical conductance (i.e., the reciprocal TEER) did not relate with para-cellular dextran-permeability, but after occurrence of a TEER-maximum, dextran-permeability and conductance increased linearly, indicative of more severe epithelial layer damage. Within 24 h after the occurrence of a TEER maximum, TEER decreased to below the level of unchallenged epithelial layers demonstrating microscopically observable holes and apoptosis. Under probiotic protection by adhering bifidobacteria, TEER-maxima were delayed or decreased in magnitude due to later transition from mild to severe damage, but similar linear relations between conductance and dextran permeability were observed as in absence of adhering bifidobacteria. Based on the time-dependence of the TEER and the relation between conductance and dextran-permeability, it is proposed that bacterial adhesion to epithelial layers first causes mild damage, followed by more severe damage after the occurrence of a TEER-maximum. The mild damage caused by *E. coli* prior to the occurrence of TEER maxima was reversible upon antibiotic treatment, but the severe damage after occurrence of TEER maxima could not be reverted by antibiotic treatment. Thus, single-time TEER is interpretable in two ways, depending whether increasing to or decreasing from its maximum. Adhering bifidobacteria elongate the time-window available for antibiotic treatment to repair initial pathogen damage to intestinal epithelial layers.

## Introduction

Trans-epithelial electrical resistance (TEER) measurements constitute a simple, non-invasive method to monitor the barrier integrity of epithelial or endothelial cell layers (Chen et al., 2015). The electrical resistances comprised in an epithelial or endothelial cell layer involve most notably the resistances of the apical and basolateral cell membranes and the intra-cellular fluid (the trans-cellular pathway) in series. These serial resistances operate in parallel with the resistance of the extra-cellular fluid contained in the tight-junctions (Suzuki et al., 2017) between cells (the para-cellular pathway) (Benson et al., 2013; Odijk et al., 2015). Since tight-junctions not only contain extra-cellular fluid but also a variety of tight-junction proteins acting as a bridge between neighboring cells, electrical current flows equally through the trans-cellular and the para-cellular pathway (Krug et al., 2009). TEER therewith reflects the integrity of the cell layer and its barrier function (Butt et al., 1990; Lippmann et al., 2012; Béduneau et al., 2014; Van der Helm et al., 2016). For non-invasive measurements of the integrity of mono-culture cell layer, TEER measurements constitute the “gold standard” (Maherally et al., 2018).

The barrier function of tight-junctions regulates host nutrition and waste removal (Groschwitz and Hogan, 2009), maintenance of homeostasis (Gareau et al., 2010) and protection of the host against pathogen invasion, such as by *Escherichia coli* that can cause severe intestinal infection (LeBlanc, 2003). At the same time, human intestinal epithelial layers are colonized by a large number of commensal bacteria, offering protection against pathogen colonization and invasion. In case the delicate balance of the gut microflora is disrupted and pathogens start to colonize, disease results (Ouwehand et al., 2016). The increasing development of antibiotic resistance amongst many pathogens makes eradication of intestinal pathogens using antibiotics more and more difficult, while indiscriminate use of antibiotics may not only kill pathogens but also the commensal intestinal microflora (Ouwehand et al., 2016; Wypych and Marsland, 2018).

Probiotics are defined by the World Health Organization as “live microorganisms that, when administered in adequate amounts, confer a health benefit on the host” (Hill et al., 2014). Probiotic bacteria are applied more and more for complementing the commensal microflora and the promotion of a healthy intestinal microflora. Probiotics operate through a variety of mechanisms including competitive inhibition of pathogen adhesion, pathogen displacement, production of bioactive metabolites, such as bacteriocins and biosurfactants, and modulation of epithelial barrier function (Ohland and MacNaughton, 2010; Reid et al., 2011). TEER has been frequently used to evaluate pathogen challenges and probiotic protection of intestinal epithelial layers. Whereas pathogenic *E. coli* or *Clostridium perfringens* have been commonly described to decrease TEER and expression of tight-junction proteins, such as claudin, occludin or zonula occludens-1 (ZO-1), a key tight-junction associated protein (Shinoda et al., 2016; Bhat et al., 2019), probiotic lactobacilli are known to increase TEER concurrent with increased expression of tight-junction proteins (Anderson et al., 2010; Corridoni et al., 2012; Barnett et al., 2018). Even in a heat-killed state, lactobacilli prevented intestinal epithelial layers against cytokine disruption, as concluded from TEER measurements (Zeng et al., 2016). Most studies on TEER and probiotic bacteria involve lactobacilli. Frequently however, the monitoring of TEER is stopped when probiotic protection is at its maximum (Anderson et al., 2010) and not pursued beyond. Also, bifidobacteria are known to exert probiotic effects, and lipopolysaccharide (LPS)-induced decreases in TEER could be prevented by bifidobacteria (Ling et al., 2016). There are, to our knowledge, only a few studies which demonstrate protective effects of probiotic bacteria with respect to intestinal epithelial integrity through TEER measurements in the simultaneous presence of pathogens, but most of these pertain to lactobacilli adhering on intestinal epithelial layers challenged by *E. coli* (Michail and Abernathy, 2002) or *Salmonella* (Fajdiga et al., 2006; Lépine et al., 2018). Experiments involving simultaneous probiotic and pathogen presence are clearly preferable, since, e.g., production and release of biosurfactants by probiotic strains, may interfere with pathogen colonization (Reid et al., 2011).

This study aims to propose a two-stage interpretative model of increasing and decreasing TEER of intestinal epithelial layers during a pathogenic *E. coli* challenge in the absence and presence of adhering probiotic bifidobacteria or the adsorbed biosurfactants they produce. To this end, we evaluated the TEER and dextran permeability of intestinal epithelial layers as a function of time during *E. coli* challenges. *E. coli* challenges were applied in the absence or presence of different adhering bifidobacterial strains or prior to and after adsorption of biosurfactants produced by the bifidobacteria used. In addition, the numbers of *E. coli* adhering to the intestinal epithelial cells were determined in the absence and presence of adhering bifidobacteria. Experiments were carried out *in vitro* in a transwell system using different co-cultures of intestinal epithelial layers and bacteria. Intestinal epithelial cell layers were imaged after cytoskeleton staining using confocal laser scanning microscopy (CLSM). Tight-junction associated protein staining was done to visualize ZO-1, while Annexin V-FITC staining was applied to observe apoptosis, employing fluorescence microscopy.

## Materials and Methods

### Bacterial Culturing and Harvesting

*Bifidobacterium breve* ATCC 15700, *Bifidobacterium longum* ATCC 15707, and *Bifidobacterium infantis* ATCC 15697 are all commensals of the human intestines and were purchased from American Type Culture Collection, while *E. coli* Hu 734 is a human clinical isolate. Bifidobacteria were streaked on RC (Reinforced Clostridial, Becton Dickinson, United States) agar plates from frozen stock and grown under anaerobic conditions (85% N_2_, 5% CO_2_, 10% H_2_) at 37°C for 48 h. *E. coli* was streaked on a blood agar plate and incubated at 37°C for 24 h. *E. coli* colonies grew on blood agar in absence of a clear- or greenish-colored zone around them, indicating absence of hemolytic activity of the strain (Buxton, 2005). Subsequently, one colony was transferred to RCM (Reinforced Clostridial Medium) broth for the bifidobacteria and to lysogeny broth (LB, Sigma-Aldrich, United States) for *E. coli*. Strains were cultured for 24 h after which bacteria were transferred (1:20) to fresh culture medium and grown for 18 h under the appropriate conditions. Bacteria were harvested by centrifugation for 5 min at 10,000 *g* and 10°C, washed twice with sterile PBS (phosphate buffered saline; 5 mM K_2_HPO_4_, 5 mM KH_2_PO_4_, 0.15 M NaCl, pH 7.0), and re-suspended in PBS for further use. Bacterial concentrations were determined by enumeration in a Bürker-Türk counting chamber, after which suspensions were diluted to concentrations required in an experiment.

### Inhibition of *E. coli* Growth by Bifidobacteria

In order to evaluate possible inhibitory effects of bifidobacteria on *E. coli* growth, a zone of inhibition assay was used. Briefly, a cotton swab was immersed in 10^5^ mL^–1^ *E. coli* suspension and spread on an RC agar plate. Then, a 10 μL droplet of 10^9^ mL^–1^ *B. breve*, *B. longum* or *B. infantis* suspension was added on an agar plate inoculated with *E. coli*. After anaerobic incubation at 37°C for 48 h, diameters of the inhibition zone around a droplet with suspended bifidobacteria were measured in three different directions and averaged.

### Biosurfactant Release by Bifidobacteria

Biosurfactant production and release of the three probiotic bifidobacterial strains was quantitated using axisymmetric-drop-shape-analysis-by-profile (ADSA-P) (Van der Vegt et al., 1991; Kwok et al., 1994). Briefly, a 100 μL droplet of a bifidobacterial suspension (5 × 10^9^ mL^–1^ in PBS) was put on a hydrophobic glass coverslip (Paul Marienfeld GmbH & Co. KG, Germany), and placed in a humidified enclosed chamber (Velraeds et al., 1996). The shape of the droplet was recorded as a function of time up to 2 h at room temperature. Biosurfactant release lowers the liquid surface tension and therewith causes time-dependent flattening of the droplet (Supplementary Movies 1 and 2). Assuming an axisymmetric drop shape, the surface tension of the suspension was calculated from the Laplace equation of capillarity

(1) $$\Delta P=\gamma \left(\frac{1}{R_{1}}+\frac{1}{R_{2}}\right)$$

which, γ is the liquid surface tension, *R*_1_ and *R*_2_ are the two principal radii of curvature of the droplet, and Δ*P* is the pressure difference across the interface. Bifidobacteria were considered to be biosurfactant releasing when the surface tension of the bacterial suspension droplet decreased by more than 8 mJ m^–2^ after 2 h (Van der Vegt et al., 1991).

Biosurfactants released by each of the different bifidobacterial strains were collected after culturing in RCM for 24 h, followed by 1:20 transfer into 200 mL RCM for 18 h (Chander et al., 2012). Spent culture medium was centrifuged at 10,000 *g* at 4°C for 20 min and the supernatant collected. The pH of supernatant was adjusted to 2 with 6 M hydrochloric acid and kept at 4°C overnight to precipitate lipids and proteins. Finally, supernatant was centrifuged again at 10,000 *g* at 4°C for 20 min, and the precipitate collected and dissolved in 10 mL PBS (pH 7.0) to a concentration of 11 mg mL^–1^ for further experiments. For control, freshly prepared RCM, not used for bacterial growth, was subjected to the same procedure.

### Intestinal Epithelial Cell Culturing and Harvesting

Caco-2 BBe cells (ATCC CRL-2102) are commonly used as a model of the human intestinal epithelial cells and were obtained from the American Type Culture Collection. Cells were grown in Dulbecco’s Modified Eagle Medium containing 4.5 g L^–1^ glucose (DMEM-HG, Gibco, United States) and 10% (vol/vol) fetal bovine serum (FBS, Gibco, United States) in 5% CO_2_ humidified incubator at 37°C. Cells were passaged after 80% confluency was achieved. Three milliliters EDTA-Trypsin (2.5 g L^–1^, Gibco, United States) was used for detaching cells in a T-75 flask at 37°C for 5 min. After detachment, DMEM-HG with 10% FBS was added for trypsin neutralization. Cells were collected by centrifugation at 800 *g* for 5 min. The cellular pellet was re-suspended and diluted in fresh culture medium at a concentration of 10^4^ mL^–1^ or 2 × 10^5^ mL^–1^ depending on the further experiment involved, as enumerated with an automated cell counter equipped with a 60 μm sensor (Merck Millipore, United States).

### Co-culture Experiments of Caco-2 BBe Layers With Bacteria and TEER Measurements

Caco-2 BBe cells were grown on 0.4 μm pore size poly(ethylene terephthalate) transwell inserts with a 1.13 cm^2^ membrane (Greiner Bio-One, Austria) from cells suspended in full culture medium (2 × 10^5^ cells mL^–1^, 0.5 mL) and the medium was refreshed every 2 days. From day 10 on, the integrity of the cellular monolayer was monitored from its TEER as measured using a Millicell^®^ ERS-2 meter (Millipore, United States). A stable TEER ≥ 400 Ω cm^2^, characteristic for intestinal epithelial layers grown in a transwell, was usually reached within 10–14 days. When the TEER was above 400 Ω cm^2^, the epithelial layer was exposed to 0.1 mL of bifidobacteria suspended in PBS (5 × 10^6^ mL^–1^) for 4 h to allow their adhesion, after which 0.1 mL of *E. coli* suspension in PBS was added at different concentrations (10^2^ mL^–1^, 10^4^ mL^–1^, 10^6^ mL^–1^). Next cells and bacteria were grown for 24 h at 37°C in a humidified incubator with 5% CO_2_ in co-culture medium. Co-culture medium was designed to allow optimal growth of Caco-2 BBe cells and bifidobacteria (Supplementary Figure 1). Caco-2 BBe cell layers in absence or presence of adhering bifidobacteria and/or *E. coli* challenges were used as controls. In a separate series of experiments, bifidobacterial biosurfactants dissolved in PBS were adsorbed to the Caco-2 BBe cell layer for 1 h prior to initiating an *E. coli* challenge.

TEER was measured as a function of time on three different locations of an epithelial layer and calculated using

(2) $${\text{TEER}}_{\text{layer}}=\left\{R_{\text{measured}}-R_{\text{membrane}}\right\}\times \mathrm{membrane}\mathrm{area}$$

which, TEER_layer_ (Ω cm^2^) is the TEER of an epithelial layer after subtraction of the TEER of the membrane without a cellular layer, R_measured_ (Ω) is the resistance measured of the membrane with a cellular layer and R_membrane_ is the resistance of the membrane measured in absence of a cellular layer.

### Adhesion of *E. coli* on Epithelial Cell Layers in Presence or Absence of Adhering Bifidobacteria or Adsorbed Biosurfactants

To evaluate whether bifidobacteria or isolated biosurfactants reduced the adhesion of *E. coli* on epithelial cell layer, cell layers with stable TEER ≥ 400 Ω cm^2^ were exposed to bifidobacteria (10^6^ mL^–1^) for 4 h or isolated biosurfactant (11 mg mL^–1^) for 1 h at 37°C, prior to exposure to *E. coli* (10^6^ mL^–1^). *E. coli* adhesion was measured 2 h after initiating the challenge (before the TEER maximum) and at the TEER maximum (4 h after challenge initiation). To this end, cell layers were washed five times with PBS and sonicated with 0.5 mL^–1^ PBS for 15 s to detach cells with adhering bacteria from the membrane. A serial dilution series was prepared in PBS and plated on LB agar plates and incubated at 37°C under aerobic conditions for 24 h in order to enumerate the number of colony forming *E. coli* units per cm^2^ (CFU/cm^2^) cell layer.

### Fluorescence Microscopy

#### Imaging of Epithelial Cell Layers

For the visualization of the Caco-2 BBe cytoskeleton, cells were fixed with 3.7% (wt/vol) paraformaldehyde and permeabilized with 0.5% (vol/vol) Triton X-100. Subsequently, cells were stained with Phalloidin-FITC (Sigma-Aldrich, United States, 495 nm excitation/520 nm emission) and 4′,6-diamidino-2-phenylindole dihydrochloride (DAPI; Sigma-Aldrich, United States, 364 nm excitation/454 nm emission) to visualize F-actin and nuclei, respectively. Cells were imaged using CLSM with 63× magnification objective lens and 0.5 μm depth per stack (Leica SP2, Germany). Fiji Software (Schindelin et al., 2012) was used to analyze the CLSM images. Images were taken of epithelial cell layers prior to and during *E. coli* challenges at 10^4^ mL^–1^ for 4, 8, and 24 h.

#### Visualization of Tight-Junction Associated Proteins

To visualize the effects of *E. coli* and bifidobacteria on tight-junction proteins in epithelial layers, cells were treated as described above, but after permeabilization cells were exposed to 5% BSA in PBS for 1 h at room temperature to block non-specific adsorption, washed once with PBS containing 0.1% Triton X-100 (PBST) for 5 min, and subsequently labeled with primary antibody rabbit-anti-human ZO-1 (1:200, #40-2300, Invitrogen) at 4°C overnight. Next, cells were washed twice with PBST for 5 min, and labeled with secondary antibody Rhodamine Red-X Donkey anti-Rabbit (1:100, #711-295-152, Jackson Immunolab, excitation 570 nm/emission 590 nm) for 1 h. Finally, cells were washed with PBST and PBS, each for 5 min and ZO-1 visualized employing fluorescence microscopy with the green laser (Leica DM4000, Germany).

#### Apoptosis Staining

To evaluate apoptosis in cellular layers upon *E. coli* challenges, cells were stained with Annexin V-FITC (Thermo Fisher Scientific, United States) which targets phosphatidylserine molecules translocated from the inner face of the plasma membrane to the cell surface, i.e., a sign of early apoptosis (Chen et al., 2008). To this end, cell layers were washed once with PBS, followed by washing with Annexin V-binding buffer and subsequently labeled with Annexin V-FITC (1:40, excitation 488 nm/emission 520 nm) at room temperature for 10 min. Then, cells were washed again with the binding buffer and additionally stained at room temperature for 5 min with propidium iodine (20 μg mL^–1^, excitation 535 nm/emission 617 nm) to confirm apoptosis signs (Chen et al., 2008) employing fluorescence microscopy. Propidium iodine is a nucleus stain, only entering membrane damaged cells. For comparison, cell layers were purposely brought in an apoptotic state by exposure to 60°C for 20 min prior to imaging.

### Dextran Permeability Measurements

The para-cellular permeabilities of the intestinal epithelial cell layers prior to and during *E. coli* challenges in absence or presence of probiotics were determined by measuring the transport of 4 or 10 kDa fluorescein isothiocyanate (FITC)-labeled dextran (FD4 or FD10S; Sigma-Aldrich, St. Louis, MO, United States) across the cell layer over time (Hubatsch et al., 2007). Free FITC in the FITC-dextran purchased, had been removed by multiple precipitations in ethanol yielding stable solutions, that were free of FITC not bound to dextran (De Belder and Granath, 1973). Dextrans were dissolved in DMEM-HG medium (5 mg mL^–1^) and 100 μL of a dextran solution was added to the apical surface of Caco-2 BBe cells in the transwell insert. 100 μL aliquots were taken from the DMEM-HG medium underneath the membrane after different time intervals during *E. coli* challenges up to 24 h, while replenishing with the same amount of fresh medium. Fluorescence intensities of the aliquots were measured using a fluorescence microplate reader (485 nm excitation/520 nm emission). FITC-labeled dextran transport across the cell layers was quantified using a calibration curve of fluorescence intensity as a function of FITC-labeled dextran concentration (Supplementary Figure 2).

The apparent para-cellular permeability coefficient (P_*app*_) was calculated according to Artursson and Karlsson (1991)

(3) $$P_{\text{app}}=\left(\frac{\Delta Q}{\Delta t}\right)\times \left(\frac{1}{\left(AxC_{0}\right)}\right)$$

which, Δ*Q* is the FITC-labeled dextran mass (g) transported through the cell layer within a time period Δ*t* (min), *A* is the membrane surface area (cm^2^) and *C*_0_ is the initial concentration (g mL^–1^) of FITC-labeled dextran above the apical cell surface of the epithelial cells grown on the transwell membrane.

### Statistical Analysis

All experiments were conducted in triplicate, and the results are represented as means ± standard error of the mean (SEM). Student’s *t*-test were used for two groups comparison and one- or two-way ANOVA were performed, followed with Tukey or Dunnett multiple comparison using GraphPad Prism 7.00. Significance was adapted at *p* < 0.05.

## Results

### Inhibition of *E. coli* Growth by Bifidobacteria

**FIGURE 1:**
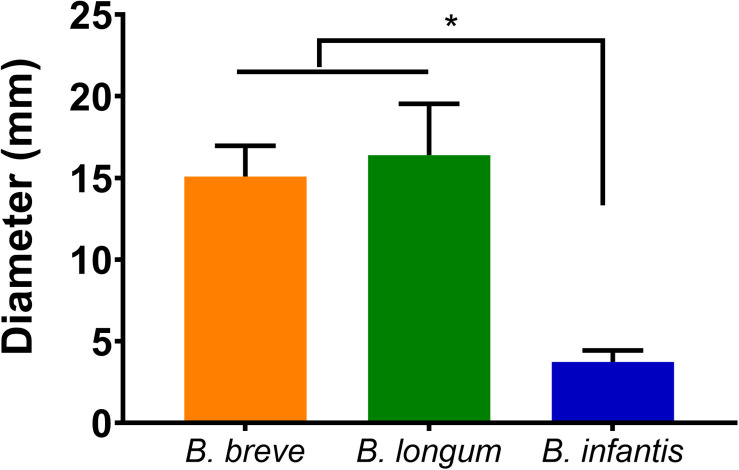
Diameter of the inhibition zones around droplets with suspended bifidobacteria: *B. breve* ATCC 15700, *B. longum* ATCC 15707, and *B. infantis* ATCC 15697 on *E. coli* Hu 734 covered agar plates. Error bars represent standard errors of the mean over three experiments with separately grown bacteria. *indicates statistical significant differences (one-way ANOVA followed with Tukey for multi-comparison, *p* < 0.05).

*Escherichia coli* growth was inhibited by *B. breve* ATCC 15700, *B. longum* ATCC 15707, and *B. infantis* ATCC 15697 as determined by zone of inhibition measurements. *B. breve* and *B. longum* exhibited significantly larger zones (*p* < 0.05, one-way ANOVA) of inhibition against *E. coli* than *B. infantis* (Figure 1).

### Biosurfactant Production by Bifidobacteria

**FIGURE 2:**
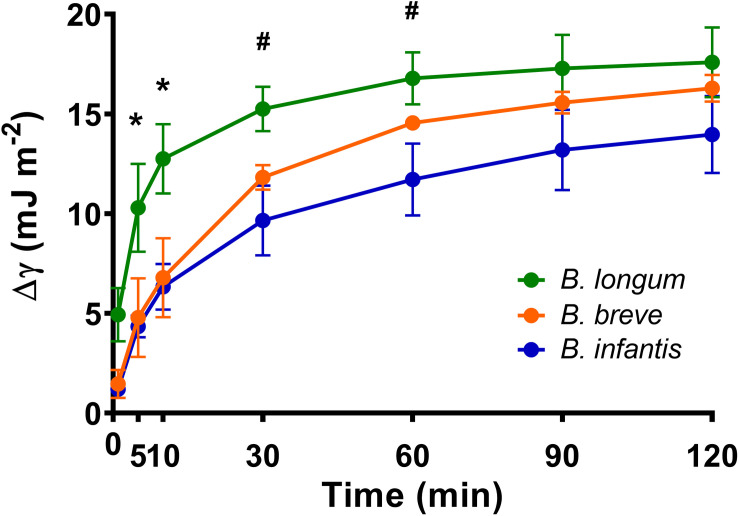
Surface tension decreases (Δγ_*lv*_) of *B. breve* ATCC 15700, *B. longum* ATCC 15707, and *B. infantis* ATCC 15697 suspensions as function of time, as an indication of biosurfactant production and release. Initial surface tensions of the bacterial suspensions in PBS amounted 67.6 ± 3.6 (mJ m^− 2^). Error bars represent standard errors of the mean over three experiments with separately grown bacteria. *indicates statistically significant differences (*p* < 0.05) between *B. longum* and both other strains, ^#^indicates statistically significant differences (*p* < 0.05) between *B. longum* and *B. infantis*.

The surface tensions of *B. breve*, *B. longum*, and *B. infantis* suspensions in PBS at t equals 0 amounted 67.6 ± 3.6 mJ m^–2^ and decreased by more than 8 mJ m^–2^ within 2 h, regardless of the strain involved (Figure 2). Considering a decrease in surface tension of more than 8 mJ m^–2^ as indicative of biosurfactant release (Van der Vegt et al., 1991), all three bifidobacterial strains can be regarded as biosurfactant releasing strains. Surface tensions of solutions of biosurfactants isolated from *B. breve, B. longum*, and *B. infantis* (11 mg mL^–1^) amounted 53.3 ± 0.7 mJ m^–2^, 52.1 ± 1.3 mJ m^–2^, and 54.7 ± 2.9 mJ m^–2^, respectively. The components isolated from fresh RCM culture medium had a higher surface tension of 61.5 ± 0.7 mJ m^–2^ than the biosurfactant solutions and suspensions of bifidobacteria after 2 h release of biosurfactants.

### Time-Dependence of the TEER of Epithelial Cell Layers Co-cultured With Bacteria

**FIGURE 3:**
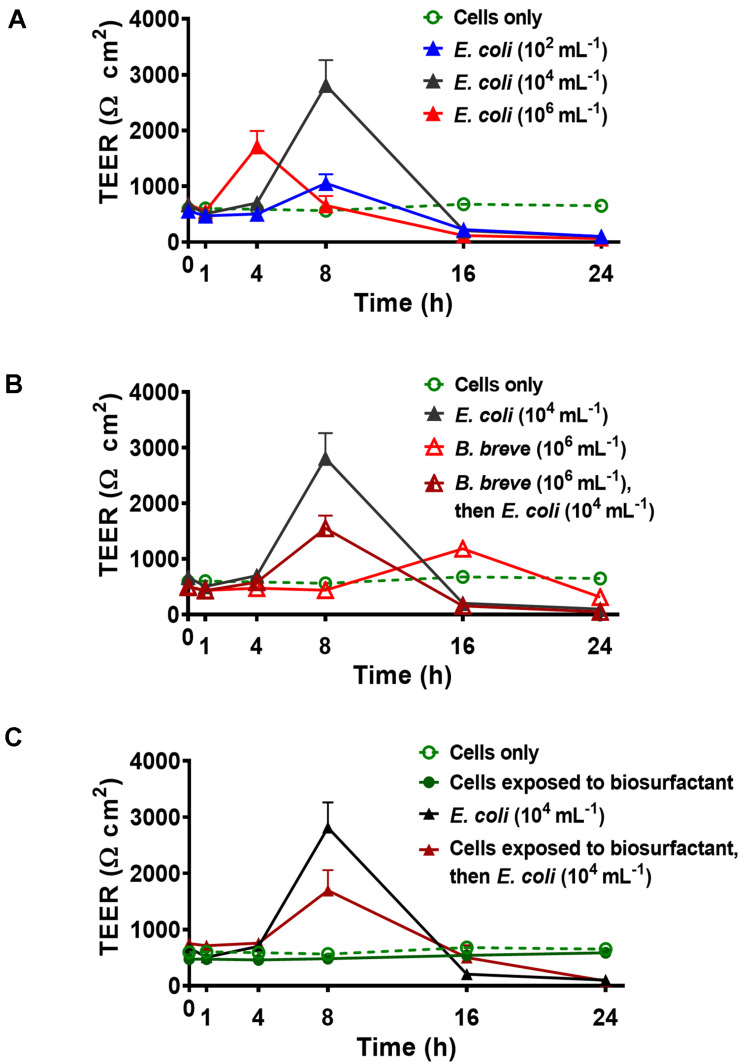
Examples of the TEER of Caco-2 BBe cell layers as a function of time upon challenging with *E. coli* Hu 734 in the absence and presence of adhering bifidobacteria or adsorbed biosurfactants. Time zero corresponds with the initiation of the *E. coli* challenge. Error bars represent standard errors of the mean over three experiments with separately grown bacteria. **(A)** TEER of Caco-2 BBe cell layers during challenges with *E. coli* at different concentrations in suspension. **(B)** TEER of Caco-2 BBe cell layers with adhering *B. breve* (4 h) and subsequently challenged with *E. coli*. **(C)** TEER of Caco-2 BBe cell layers during challenges with *E. coli* prior to and after adsorption (1 h) of *B. breve* biosurfactants, including the TEER of epithelial layers with adsorbed biosurfactants in the absence of an *E. coli* challenge.

Trans-epithelial electrical resistance of Caco-2 BBe cell layers as a function of time during an *E. coli* Hu 734 challenge in absence and presence of adhering bifidobacteria are presented in Figure 3, while quantitative features of the time-dependence of TEER are compiled in Table 1. Caco-2 BBe cell layers in absence of adhering probiotic bacteria or pathogen challenges, demonstrated a stable TEER of around 613 ± 78 Ω cm^2^. During challenging the epithelial layer with *E. coli*, TEER increased over time to reach a maximal value after 4–8 h (Figure 3A and Table 1). The TEER maximum occurred earliest at the highest *E. coli* challenge concentration (10^6^ mL^1^). For the two lower *E. coli* concentrations, the TEER maximum occurred later while resistance increased with *E. coli* concentrations up to 10^4^ mL^–1^. However, for all *E. coli* challenge concentrations, the TEER of the layer was reduced to around 100 Ω cm^2^ or less after 24 h of challenge (see Table 1), with the strongest decrease occurring at the highest *E. coli* challenge concentration (10^6^ mL^–1^). The presence of adhering probiotic *B. breve* in absence of an *E. coli* challenge on the cellular layers also yielded a TEER maximum similar as during an *E. coli* challenge, but this maximum occurred generally later and was relatively low (Figure 3B). A reduction in 24 h TEER as observed during an *E. coli* challenge was also seen, but only for adhering *B. breve* and not for *B. longum* and *B. infantis* (see Table 1).

**TABLE 1:** Summary of the time-dependence of trans-epithelial electrical resistances of Caco-2 BBe layers, challenged by different concentrations of pathogenic *E. coli* Hu 734 in the absence or presence of adhering probiotic bifidobacteria or adsorbed biosurfactants (see Figure 3 for examples).

*E. coli* concentration (mL^–^^1^)		Time to maximum (h)	Maximal TEER (Ω cm^2^)	24 h TEER (Ω cm^2^)
**No adhering probiotic bacteria/no adsorbed biosurfactants**				
0		No maximum	No maximum	61378
10^2^		8	1055 ± 160	10131^a^
10^4^		8	2814 ± 449	1016^a^
10^6^		4	1713 ± 280	629^a^
**Adhering *B. breve* ATCC 15700**				
0		16	1183 ± 26^b^	31663^a^
10^2^		16	729 ± 493	8311a
10^4^		8	1551 ± 230^b^	504^a^
10^6^		4	1229 ± 241	6013^a^
**Adhering *B. longum* ATCC 15707**				
0		No maximum	No maximum	66134
10^2^		8	620 ± 71	5722^a^
10^4^		8	1742 ± 232^b^	565^a^
10^6^		8	1380 ± 122	6420^a^
**Adhering *B. infantis* ATCC 15697**				
0		No maximum	No maximum	749136
10^2^		8	764 ± 138	4911^a^
10^4^		8	1405 ± 145^b^	7711^a^
10^6^		8	1220 ± 261	6716^a^
**Adsorbed biosurfactants only**				
0	*B. breve*	No maximum	No maximum	58441
	*B. longum*	No maximum	No maximum	62821
	*B. infantis*	No maximum	No maximum	6564
10^4^	*B. breve*	8	1695 ± 362^b^	8210^a^
	*B. longum*	8	1583 ± 36^b^	803^a^
	*B. infantis*	8	1998 ± 138^b^	817^a^

*All data are expressed as means ± standard error of the mean over three different experiments with separately grown cellular layers and bacteria.^a^Significant differences in TEER at p < 0.05 with respect to cellular layers grown in the absence of adhering probiotic bacteria and E. coli challenges.^b^Statistically significant differences between the absence and presence of adhering probiotic bacteria at corresponding E. coli concentrations.*

The presence of adhering bifidobacteria affected the effects of pathogenic *E. coli* challenge in two ways, depending on the probiotic strain involved (Figure 3B and Table 1): (1) it delayed the development of a TEER maximum due to the *E. coli* challenge, and/or (2) it reduced the value of the TEER maximum. However, adhering bifidobacteria could not prevent the reduction in TEER after 24 h exposure to an *E. coli* challenge (Table 1).

Trans-epithelial electrical resistance of epithelial layers exposed to biosurfactant solutions did not increase over time, and by consequence the TEER values did not show a maximum over the experimental period (Figure 3C and Table 1). Additionally, the 24 h TEER value was similar as of an unchallenged epithelial layer (Table 1). During an *E. coli* challenge, epithelial layers with adsorbed biosurfactants demonstrated a similarly low TEER maximum when challenged with *E. coli* in the presence of adhering bifidobacteria (Figure 3C and Table 1). Like adhering bifidobacteria, adsorbed biosurfactant*s* could not prevent a TEER decrease to below the level of untreated cell layers after a 24 h challenge with *E. coli* (Table 1).

### Adhesion of *E. coli* on the Epithelial Cell Layers

**FIGURE 4:**
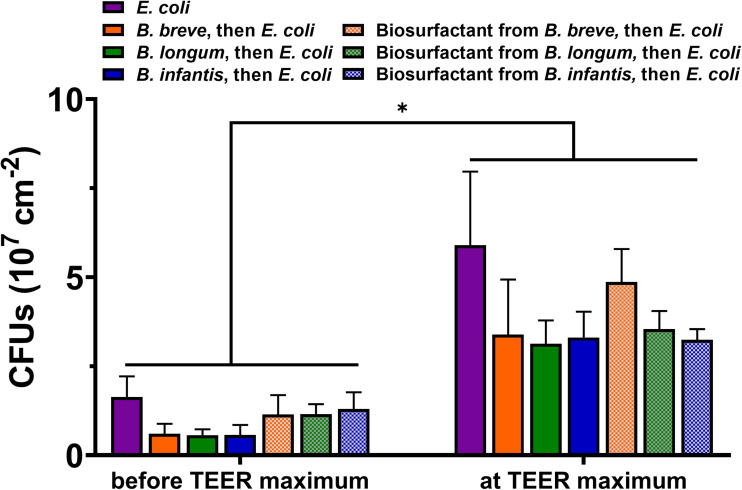
Adhesion of *E. coli* Hu 734 on intestinal epithelial cell layers in the absence or presence of adhering *B. breve* ATCC 15700, *B. longum* ATCC 15707, *B. infantis* ATCC 15697 or their adsorbed biosurfactants. *E. coli* adhesion was enumerated 2 h after initiating *E. coli* adhesion (before the TEER maximum occurred) and at the TEER maximum (4 h after initiating *E. coli* adhesion). PBS was used as a control. Error bars represent standard errors of the mean over three experiments with separately grown cellular layers and bacteria. *indicates statistically significant difference in CFUs before the occurrence of TEER maximum and at the TEER maximum.

The number of adhering of *E. coli* per unit area on the epithelial cell layers increased significantly (*p* < 0.05) over time toward the occurrence of the TEER maximum, regardless of the absence or presence of adhering bifidobacteria or the adsorbed biosurfactants they produce (Figure 4). Protection of the cell layers by adhering bifidobacteria against *E. coli* adhesion can be seen both 2 and 4 h after initiating *E. coli* adhesion. Probiotic adhesion caused greater reductions in *E. coli* adhesion than adsorbed biosurfactants, although this was not statistically significant.

### Visualization of Cell Layers Prior to and After an *E. coli* Challenge

**FIGURE 5:**
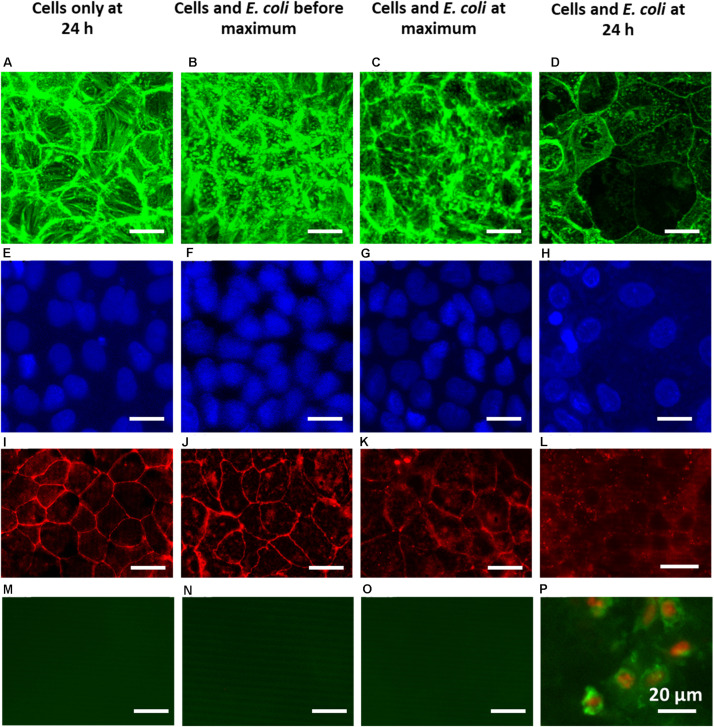
Overlayer images of confocal stacks of Caco-2 BBe cell layers showing green-fluorescent cytoskeleton **(A–D)** and blue-fluorescent nuclei **(E–H)**, fluorescence images of red-fluorescent ZO-1 tight-junction proteins **(I–L)** and apoptosis of green-fluorescent membrane-damaged cells with red-fluorescent nuclei **(M–P)**. For comparison, a fluorescent image of apoptotic cells is shown in Supplementary Figure 3. **(A,E,I,M)** 24 h Caco-2 BBe cell layers grown in the absence of an *E. coli* challenge. **(B,F,J,N)** Caco-2 BBe cell layers in the presence of an *E. coli* (10^4^ mL^− 1^) challenge before the occurrence of the TEER maximum, i.e., at 4 h. **(C,G,K,O)** Caco-2 BBe cell layers grown in the presence of an *E. coli* (10^4^ mL^− 1^) challenge at the TEER maximum, i.e., at 8 h. **(D,H,L,P)** Caco-2 BBe cell layers grown in the presence of an *E. coli* (10^4^ mL^− 1^) challenge after the occurrence of the TEER maximum, i.e., after 24 h.

F-actin and nucleus staining of epithelial cell layers showed a dense network of cells (Figures 5A,E), held together by clearly visible tight-junction proteins (Figure 5I). During an *E. coli* challenge, the number of cells and cytoskeleton (Figures 5B,F), as well as tight-junction proteins connecting neighboring cells (Figure 5J), remained roughly similar as before challenge in the first 4 h. At the TEER maximum, the layer structure was less dense (Figure 5C) than before an *E. coli* challenge, as can be seen from both the F-actin (Figure 5C) and nucleus images (Figure 5G). Thus, at the TEER maximum, mild damage to the epithelial layer had developed. At the same time, tight-junction proteins were still present outlining the circumference of all cells, but with a more “fuzzy” red-fluorescence rim than in cell layers in absence of an *E. coli* challenge (compare Figure 5K and Figure 5I). This likely indicates scattered increased expression of tight-junction protein ZO-1. After 24 h of challenge, i.e., well after the TEER maximum occurred, large black holes were visible (Figure 5D), with a decreased number of nuclei (Figure 5H). Furthermore, tight-junction proteins were no longer fully outlining the circumference of each cell (Figure 5L), illustrating severe damage to the integrity of the epithelial cell layer. Apoptotic cells were only observed after 24 h *E. coli* challenges (Figure 5P) and not for shorter challenge times prior to the TEER maximum (Figures 5N,O), indicating that apoptosis only occurred after the TEER maximum.

### Para-Cellular Permeability by Dextran

**FIGURE 6:**
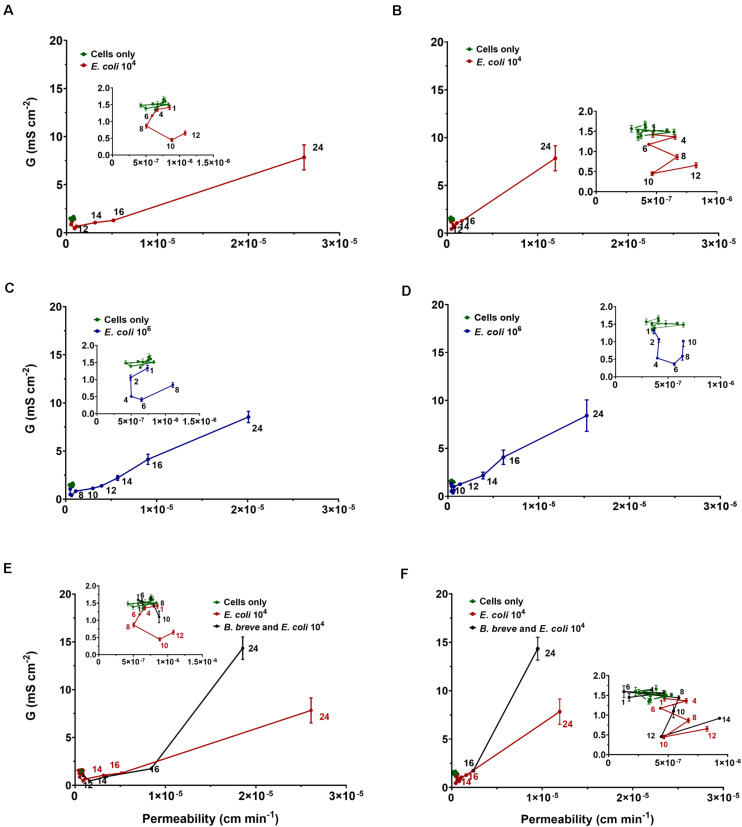
The conductance G (reciprocal TEER) of Caco-2 BBe cell layers at different times after initiating an *E. coli* challenge in the absence and presence of *B. breve* ATCC 15700 (10^6^ mL^− 1^) as a function of their permeability. The insets detail conductance as a function of permeability prior to the onset of linearity. **(A)** Conductance of epithelial cell layers as a function of permeability for 4 kDa FITC-labeled dextran at an *E. coli* challenge of (10^4^ mL^− 1^). **(B)** Same as panel a, now for 10 kDa FITC-labeled dextran. **(C)** Conductance of epithelial cell layers as a function of permeability for 4 kDa FITC-labeled dextran at an *E. coli* challenge of (10^6^ mL^− 1^). **(D)** Same as panel c, now for 10 kDa FITC-labeled dextran. **(E)** Conductance of epithelial cell layers as a function of permeability for 4 kDa FITC-labeled dextran at *E. coli* challenge (10^4^ mL^− 1^) in absence and presence of *B. breve* (10^6^ mL^− 1^). **(F)** Same as panel e, now for 10 kDa FITC-labeled dextran. Error bars represent standard errors of the mean over three experiments with separately grown cellular layers and bacteria.

Mass transport of 4 and 10 kDa dextran increased linearly over time through intestinal epithelial layers in the absence of an *E. coli* challenge or probiotic protection (Supplementary Figure 4). Permeabilities calculated from the FITC-dextran transport upon *E. coli* challenges are presented in Figure 6 as a function of the conductance, i.e., the reciprocal TEER, indicative of ion transport through the cell layers. In absence of an *E. coli* challenge, 4 kDa dextran demonstrated slightly (1.5×) but significantly (*p* < 0.01, *t*-test) higher permeabilities than 10 kDa dextran. Permeabilities were relatively stable up to 8–12 h under *E. coli* challenges (Figures 6A,B) while electrical conductance was decreasing due to bacterial adhesion. After a minimum in conductance, i.e., the maximum in TEER, dextran permeability increased linearly with conductance, suggesting that transported ions use the same para-cellular pathway through an intestinal epithelial layer as dextran, irrespective of its molecular weight. Thus barrier damage has become more severe. In line with the data in Table 1, epithelial layers challenged with a higher (10^6^ mL^–1^) concentration of *E. coli* (Figures 6C,D) demonstrated a minimum conductance after a shorter exposure time (i.e., 6 h) followed by a linear trajectory at longer exposure times. Under probiotic protection, similar relations between permeability and conductance were observed (Figures 6E,F) as in absence of adhering bifidobacteria, but with a delayed occurrence of the transition from mild to more severe epithelial layer damage, characterized by the on-set of linearity between conductance and permeability.

## Discussion

Intestinal epithelial layers were challenged by *E. coli* in the absence or presence of different adhering bifidobacterial strains. Unchallenged intestinal epithelial cell layers grown in a transwell had a TEER value of 613 Ω cm^2^, in agreement with literature data on Caco-2 cell layers (Odijk et al., 2015) and considered representative of intestinal barrier integrity.

*Escherichia coli* challenges led to an increase in TEER within 4–8 h and resistance depended on the *E. coli* challenge concentration and the number *E. coli* adhering to the epithelial cell layers (Table 1 and Figure 4). The increase in TEER to a maximal resistance upon pathogen challenges is due to a combination of factors. Firstly, the number of bacteria adhering to the epithelial cell layer increases, yielding an additional resistance to the TEER of the cell layer, while also adhering pathogens can down regulate cellular ion transporters, contributing to a higher TEER (Turner et al., 2000; Gill et al., 2007; Hodges et al., 2008; Das et al., 2018). Secondly, during the period of increasing TEER, a clear “fuzzy” red-fluorescence rim indicative of ZO-1 expression developed around epithelial cells in the layer upon pathogen challenge (Figures 5I–L). Such a “fuzzy” coat was less clearly observed in absence of an *E. coli* challenge, which may imply scattered, increased expression of the tight-junction protein ZO-1 of cells under pathogen challenge. This is in line with the known stimulation of integrin-expression in mammalian cells by low level pathogen challenges to allow them to adhere more intimately to surfaces (Kim et al., 2009; Engels-Deutsch et al., 2011; Yue et al., 2015). Microscopically, the cell layer became less densely structured, although no indication of apoptotic processes was seen (Figure 5). During the time period that TEER increased to a maximal value, a clear relation between transport of ions (i.e., conductance) and changing dextran permeability was lacking (Figure 6). Collectively, this suggests that the increase in TEER toward its maximum is a result of bacterial adhesion to the epithelial cell layer, increased expression of tight-junction proteins, most notably ZO-1 and mild damage to the epithelial layer. Importantly, the damage to epithelial layers occurring prior to the TEER maximum is reversible upon antibiotic treatment (see Supplementary Figure 5).

Once TEER had reached a maximum upon an *E. coli* challenge, it decreased to below the level of an unchallenged epithelial layer, concurrent with microscopically observable severe damage, including holes in the epithelial layer and apoptosis due to bacterial toxins (Turner et al., 2000; Gill et al., 2007; Hodges et al., 2008; Das et al., 2018). This damage also widened up the tight-junctions and caused cell dissociation, providing a low resistance para-cellular pathway for electrical current after the occurrence of the TEER maximum, characteristic of what has been dubbed as “leaky” epithelial (Troeger et al., 2007b). The linear relation between conductance and dextran permeability supports bacterial widening of tight-junctions, not only allowing increased transport of ions but also of dextran. Pathogenic bacteria possess a wide array of mechanisms that can either affect the epithelial cytoskeleton (de Souza Santos and Orth, 2015) or even fully breakdown tight-junctions and epithelial cell layers due to secretion of toxins (Turner et al., 2000; Gill et al., 2007; Hodges et al., 2008; Franco and Shuman, 2012; Das et al., 2018), in line with the course of TEER and the relation between conductance and dextran permeability over time observed here. Severely damaged epithelial layers at or after the occurrence of the TEER maximum could not be reverted upon antibiotic treatment (see also Supplementary Figure 5).

**FIGURE 7:**
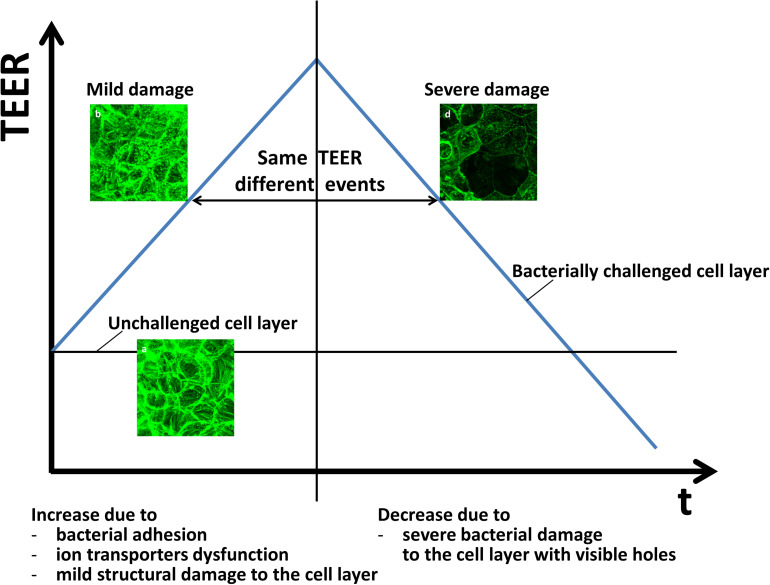
Two-stage damage model to the intestinal epithelial layers during probiotic protection and pathogen challenge. Error bars represent standard errors of the mean over three experiments with separately grown bacteria and cells.

Adhesion of *B. breve* in absence of *E. coli* challenges led to a delayed and far lower TEER maximum than observed during an *E. coli* challenge, occurring only after 16 h. Adhesion of *B. longum* and *B. infantis* did not lead to a TEER maximum. Absence of a TEER maximum in case of adhering probiotic bacteria may have two reasons: (1) since permanent instillation of probiotic bacteria in the gut is usually troublesome (Williams, 2010; Zmora et al., 2018), this attests to their low adhesiveness (Zmora et al., 2018); (2) the “healthy” character of probiotic bacteria may be accompanied by an inability to stimulate integrin-expression in the epithelial layer to the same extent as a pathogen might do (Wang et al., 2006; Parolin et al., 2018). In addition, 24 h adhesion of the probiotic strains did not result in a strong decrease in TEER to below the level of an unchallenged epithelial layer as caused by a pathogenic *E. coli* strain (*B. breve* did cause a small but significant decrease in 24 h TEER, but not to the low level observed for *E. coli* only). Other probiotic strains than bifidobacteria have been found before to maintain or enhance epithelial barrier integrity, i.e., maintaining a stable or increased TEER, during short co-culture times (Ramos et al., 2013), while pathogenic strains more readily damaged barrier integrity of epithelial layers (Hasan et al., 2018). Adsorbed biosurfactant protected epithelial layers according to a similar TEER response as observed when bifidobacteria were adhering on the epithelial layers during *E. coli* challenges. This confirms the crucial role of biosurfactants in probiotic action (Rivardo et al., 2009; Sharma and Saharan, 2014). In the present study, it will likely reflect the ability of adsorbed biosurfactants to protect a surface against pathogen adhesion (see also Figure 4; Velraeds et al., 1996, 1997, 1998, 2000; Busscher et al., 1997; Van Hoogmoed et al., 2004; Rivardo et al., 2009). Also in a TEER-based study, bioactive metabolites (“cell-free supernatant”) of *Bifidobacterium lactis* protected Caco-2 epithelial junctions against *E. coli* (Putaala et al., 2008), which is in line with protection offered by adsorbed biosurfactants in this study. However, the study of Putaala et al. (2008) was done with bioactive metabolites that were not identified as possessing biosurfactants. The time-dependent changes observed in the TEER of intestinal epithelial layers during probiotic protection and pathogen challenge can be interpreted on the basis of a two-stage damage model to the cell layer (Figure 7). In the model, adhering bacteria are assumed to initially increase TEER due to their adhesion to the cellular layer directly providing an additional resistance, stimulation of tight-junction protein expression and dysfunctioning of cellular ions transporters. At the same time, bacterial toxins cause mild damage to the epithelial layer. Mild damage is characterized by a less dense structure of the cell layer, in absence of a relation between ion transport, i.e., conductance and dextran permeability.

After longer exposure times, marked by the appearance of a TEER maximum, pathogen challenges cause more severe damage to the cell layer, including apoptosis, widening of tight-junctions and creation of holes that taken together decreases the TEER to below the TEER of an unchallenged epithelial layer. In this severe damage stage, transport of ions (conductance) and dextran (permeability) are linearly related. Probiotic bifidobacteria did not demonstrate this course of events and moreover, all bifidobacterial strains in our study reduced the negative impact of a pathogenic *E. coli* strain on epithelial barrier function, as evidence by a delayed appearance of the second damage stage to the epithelial layer. Therefore, this two-stage damage model provides a more extensive way to explain biological events in an epithelial cell layer during simultaneous probiotic protection and pathogen challenge (Figure 7). Adhesion of probiotic bacteria protects epithelial layers against damage by adhering pathogenic *E. coli* is reflected by a delayed occurrence of a lower TEER maximum. However, eventually upon long-term exposure, both probiotic bacteria and pathogenic bacteria may cause damage to the epithelial barrier integrity, as evidenced by a strongly reduced TEER. This is a common observation *in vitro*, both for probiotic bacteria (Kim et al., 2012) as well as for pathogenic strains (Hasan et al., 2018). This may reflect that overdosing of probiotics as a daily intake should be avoided and may lead to diarrhea (Boyle et al., 2006; Williams, 2010). However, the relatively short time period over which both probiotic and pathogenic bacteria cause damage to the epithelial barrier integrity *in vitro* is not reflecting the *in vivo* situation adequately (Deng et al., 2018). *In vivo*, cellular turnover, which is not included in our *in vitro* model employed, will delay the occurrence of these complications.

Our two-stage interpretation of changes in TEER of intestinal epithelial layers, will extend to other pathogens than *E. coli*, including protozoa (Li et al., 1994; Troeger et al., 2007a) and also encompassing the *in vivo* situation (Alaish et al., 2013), for which similar decreases in TEER (Deng et al., 2018; Malago et al., 2003) and increased dextran permeabilities (Alaish et al., 2013) have been described. However, due to difference in virulence between pathogens and the complexity of the *in vivo* situation, the time-frame of the different stages distinguished on the basis of changes TEER may be different. In murine models for example, apoptosis due to a *Clostridium difficile* pathogen challenge occurred already within 2 h (Deng et al., 2018).

Concluding, for proper interpretation of TEER readings and description of the status of cell layers, single-time point reading of TEER is clearly insufficient to describe changes in the epithelial layer and tight-junctions. Events prior to and after the appearance of a maximal TEER are distinctly different, depending whether measured when TEER is increasing toward its maximum or decreasing from it. Moreover, antibiotic treatment could not revert the severe damage to epithelial layers after the occurrence of a TEER maximum. Since probiotic protection delays or inhibits the formation of the TEER maximum depending on the probiotic strain used, probiotics thus elongates the time-window for effective antibiotic treatment of infected intestinal epithelium.

## Data Availability Statement

The raw data supporting the conclusions of this article will be made available by the authors, without undue reservation.

## Author Contributions

LY carried out the experiments under the daily supervision of BP. All authors designed the experiments, analyzed the data, and contributed to manuscript preparation.

## Conflict of Interest

HB is director of a consulting company SASA BV. The remaining authors declare that the research was conducted in the absence of any commercial or financial relationships that could be construed as a potential conflict of interest. Opinions and assertions contained herein are those of the authors and are not construed as necessarily representing views of the funding organization or their respective employer(s).
