# Supplementary material for: Two-Stage Interpretation of Changes in TEER of Intestinal Epithelial Layers Protected by Adhering Bifidobacteria During E. coli Challenges
Source: Front Microbiol. 2020 Nov 19;11:599555. doi: 10.3389/fmicb.2020.599555 (PMC7710611; doi:10.3389/fmicb.2020.599555)
Supplement: Supplementary file 1 [file Data_Sheet_1.DOCX]

Supplementary Material

## 1. Supplementary Method:

Co-culture experiments require a medium in which both bacteria and mammalian cells can grow (Subbiahdoss et al., 2009). In order to grow both bifidobacteria and Caco-2 BBe cells simultaneously, Caco-2 BBe cell culture medium (DMEM-HG + 10% FBS) was supplemented with different percentages of bifidobacteria culture medium (RCM). Bifidobacteria were cultured in the differently composed co-culture media at an initial concentration of 10^7^ mL^-1^ in 5% CO_2_, 37°C and incubated for 24 h after which the number of colony-forming units (CFU) in the medium was quantified by plating serial dilutions of the bacterial suspension on RC agar plates. Enumeration was done after 24 h of growth. Caco-2 BBe cells were seeded from full medium (10^4^ mL^-1^) in a 24 wells plate and medium refreshed every other day. After 3 days, differently composed co-culture media were added and cells were grown for an additional 24 or 72 h. Co-culture media were refreshed at the day 2. A MTT assay (Mosmann, 1983) was used for evaluating the metabolic activity of the cells in co-culture media. Briefly, 100 µL of 3-(4,5-dimethylthiazol-2-yl)-2,5-diphenyltetrazolium bromide (5 mg mL^-1^) was added to medium for 4 h incubation. The formazan product was dissolved in 1 mL DMSO and optical density read at 560 nm. In addition, phase contrast microscopy was applied to observe the morphology of the cells.

Co-culture medium with Caco-2 BBe cell metabolic activity above 90% and no abnormal morphology, and yielding more than 7 CFU log-units after 24 h growth for all three bifidobacterial strains, was taken as the co-culture medium for further use in this study, which is 30% RCM medium mixed with 70% full cell culture medium.

## 2. Supplementary Figures

**
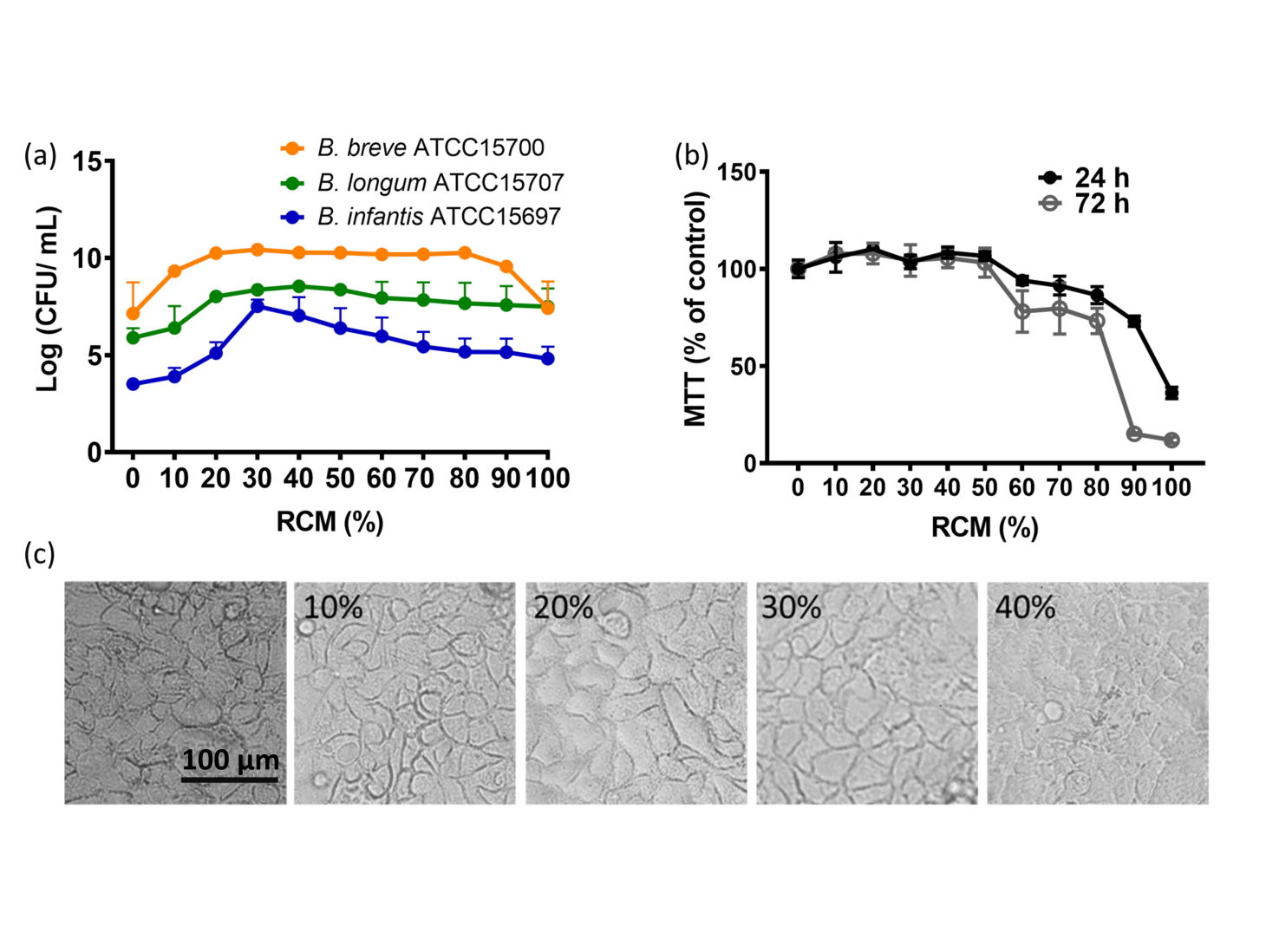
**

**Supplementary Figure S1.** Development of a co-culture medium for the simultaneous growth of bifidobacteria and Caco-2 BBe cells.

**a.** The number of bifidobacterial CFUs as a function of the percentage of RCM in DMEM-HG+FBS after 24 h of growth. Error bars represent standard errors of the mean over three experiments with separately grown bacteria.

**b.** %MTT conversion by Caco-2 BBe cells as a function of the percentage of RCM in DMEM-HG+FBS after 24 h and 72 h of growth, relative to growth in 100% DMEM-HG+FBS. Error bars represent standard errors of the mean over three experiments with separately grown cellular layers.

**c.** Phase-contrast images of Caco-2 BBe cells cultured in DMEM-HG+FBS with different percentages of RCM added.


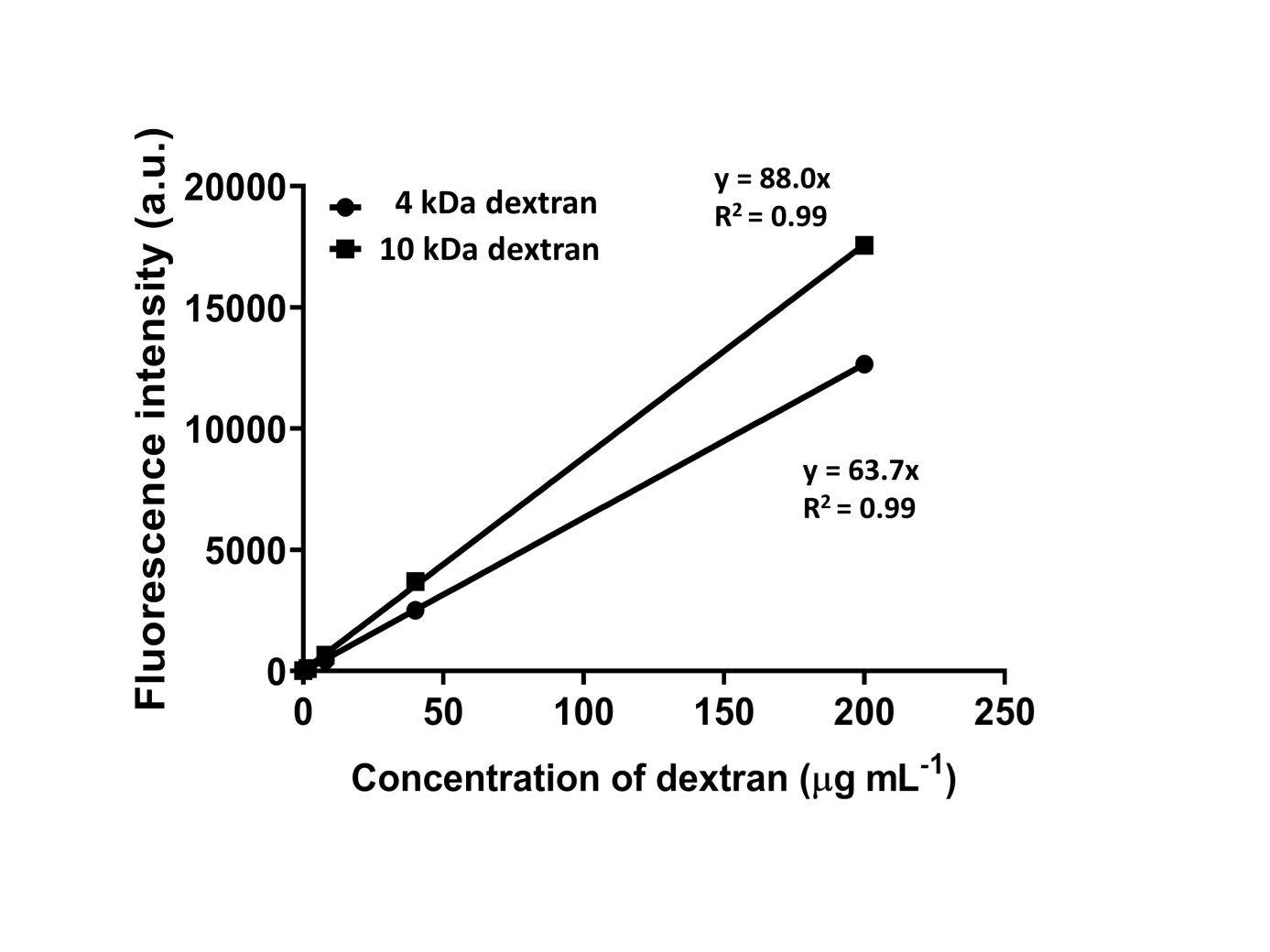


**Supplementary Figure S2.** Fluorescence intensity as a function of 4 and 10 kDa FITC-labeled dextran concentration (485 nm excitation/520 nm emission).


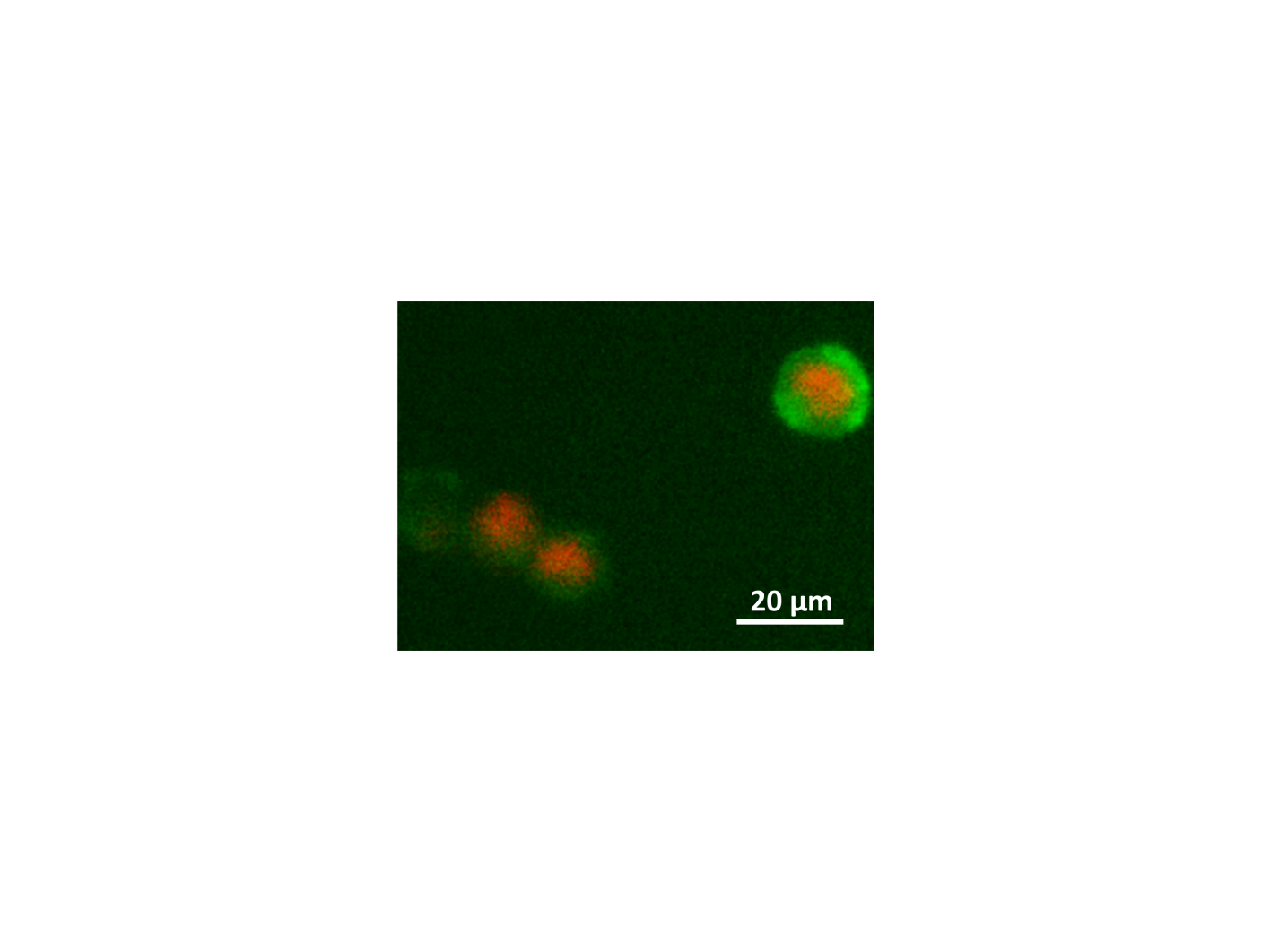


**Supplementary Figure S3.** Fluorescence image of Caco-2 BBe cells, purposely made apoptotic by exposure to 60°C for 20 min and stained with Annexin V-FITC/PI.


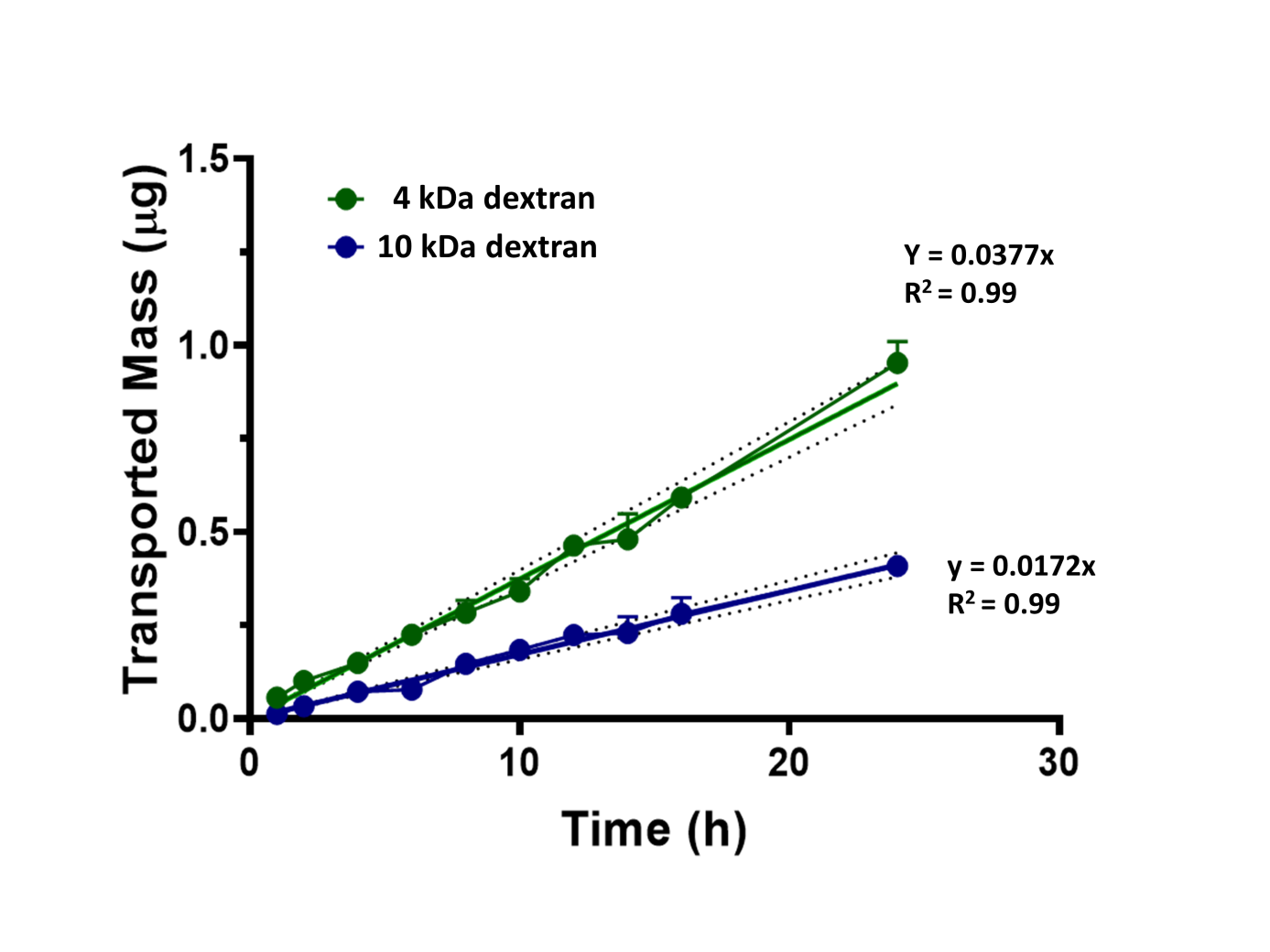


**Supplementary Figure S4.** Cumulative mass transported of 4 and 10 kDa FITC-labeled dextran across Caco-2 BBe cells layers, including an assumed linear relation demonstrating a good quality of the fit. Error bars represent standard errors of the mean over three experiments with separately grown cellular layers.

**Supplementary Figure S5.** TEER of epithelial cell layers upon an *E. coli* Hu 734 challenge (10^6^ mL^-1^) in absence and presence of tobramycin (320 μg mL^‑1^) treatment. Tobramycin treatment was initiated different times after initiating the pathogens challenge, i.e. before the occurrence of the TEER maximum and at the TEER maximum. Only antibiotic treatment initiated before the occurrence of the TEER maximum could revert the TEER back to the value observed for a healthy intestinal epithelial layer. Error bars represent standard errors of the mean over three experiments with separately grown cellular layers and bacteria.

## 3. Supplementary Movies and Captions

**Movie S1.** A time-lapse video recording of PBS (100 µL) droplet on hydrophobic cover glass over 2 h. The series of images were also used for surface tension analysis.

**Movie S2.** A time-lapse video recording of the shape of *B. longum* suspension (5 × 10^9^ mL^-1^, 100 µL) droplet on hydrophobic cover glass over 2 h, demonstrating the surfactive nature of the bioactive metabolites released by *B. longum*. The series of images were also used for surface tension analysis.

## 4. Supplementary References

Mosmann, T. (1983). Rapid colorimetric assay for cellular growth and survival: Application to proliferation and cytotoxicity assays. *Journal of Immunological Methods*, 65, 55–63. doi:10.1016/0022-1759(83)90303-4

Subbiahdoss, G., Kuijer, R., Grijpma, D. W., van der Mei, H. C., & Busscher, H. J. (2009). Microbial biofilm growth vs. tissue integration: “The race for the surface” experimentally studied. *Acta Biomaterialia,* 5(5), 1399-1404. doi:10.1016/j.actbio.2008.12.011
